# Impact of Multifocality on the Recurrence of Papillary Thyroid Carcinoma

**DOI:** 10.3390/jcm10215144

**Published:** 2021-11-02

**Authors:** Joohyun Woo, Hyeonkyeong Kim, Hyungju Kwon

**Affiliations:** Department of Surgery, Ewha Womans University Medical Center, 1071 Anyangcheon-ro, Yangcheon-Gu, Seoul 07985, Korea; jwoo@ewha.ac.kr (J.W.); cathy6280@naver.com (H.K.)

**Keywords:** papillary thyroid carcinoma, multifocality, recurrence

## Abstract

The incidence of thyroid cancer has dramatically increased over the last few decades, and up to 60% of patients have multifocal tumors. However, the prognostic impact of multifocality in patients with papillary thyroid carcinoma (PTC) remains unestablished and controversial. We evaluate whether multifocality can predict the recurrence of PTC. A total of 1249 patients who underwent total thyroidectomy for PTC at the Ewha Medical Center between March 2012 and December 2019 were reviewed. In this study, multifocality was found in 487 patients (39.0%) and the mean follow-up period was 5.5 ± 2.7 years. Multifocality was associated with high-risk features for recurrence, including extrathyroidal extension, lymph node metastasis, and margin involvement. After adjustment of those clinicopathological features, 10-year disease-free survival was 93.3% in patients with multifocal tumors, whereas those with unifocal disease showed 97.6% (*p* = 0.011). Multivariate Cox regression analysis indicated that male sex (HR 2.185, 95% CI 1.047–4.559), tumor size (HR 1.806, 95% CI 1.337–2.441), N1b LN metastasis (HR 3.603, 95% CI 1.207–10.757), and multifocality (HR 1.986, 95% CI 1.015–3.888) were independent predictors of recurrence. In conclusion, multifocality increased the risk of recurrence in patients with PTC. Patients with multifocal PTCs may need judicious treatment and follow-up approaches.

## 1. Introduction

Thyroid cancer is one of the more common cancers worldwide, and its incidence has rapidly increased over the last few decades [1]. In 2018, 567,233 patients were newly diagnosed with thyroid cancer, accounting for 3.1% of total cancer cases. Papillary thyroid carcinoma (PTC) represents more than 80% of all thyroid malignancies, which usually have a favorable prognosis [2]. Nevertheless, up to 50% of patients experience cancer relapse, including loco-regional recurrences or distant metastases [2,3]. Many studies have attempted to differentiate these patients at high risk from the population with excellent outcomes [4,5]. Several clinicopathological factors, including tumor size, extrathyroidal extension (ETE), and multifocality of tumor have been investigated to predict recurrence.

Multifocality has been considered as a prognostic marker for the recurrence of PTC [6,7]. The latest American Thyroid Association (ATA) and the European Thyroid Association (ETA) guidelines included multifocality as a risk factor for recurrence [8,9]. Although multifocality alone without other risk factors was classified as a low-risk category, these guidelines indicated that multifocality could assist in proper risk stratification for predicting recurrence. A consensus report of the European Society of Endocrine Surgeons suggested that multifocality might have a prognostic impact in overt PTC [6]. Other risk stratification systems also addressed the prognostic role of multifocality on cancer-specific survival [10]. However, recent studies raised questions about the impact of multifocality on the recurrence of PTC [11,12].

There is a controversy about the prognostic significance of multifocality in PTC. Several studies have suggested that multifocality is associated with a higher risk of recurrences and distant metastasis [13,14,15]. A few researchers further demonstrated that multifocal PTCs could decrease overall and cancer-specific survival [16]. On the contrary, other research has indicated that patients with multifocal disease showed a similar clinical course or comparable recurrence rates to those with unifocal disease [17,18]. A large multicenter study also suggested that multifocality of PTC had no independent impact on recurrences and mortality after adjustment of potential confounders [12]. Lim et al. further reported that patients with multifocal diseases might have lower risk of recurrences [19]. These conflicting data resulted from, at least in part, unadjusted clinicopathological characteristics or a limited number of patients.

In the present study, therefore, we investigated the effect of multifocality to the recurrence of PTC in a large cohort, using propensity score matching for adjustment of confounders.

## 2. Materials and Methods

Our institutional review board approved this retrospective cohort study (Approval No. 2021-07-015) and waived the requirement for written informed consent. This study included 1249 consecutive patients with thyroid cancer who underwent total thyroidectomy from March 2012 to December 2019. Neck ultrasonography and computed tomography was performed preoperatively in all patients to evaluate tumor location, multifocality, and cervical lymph node (LN) metastasis. Patients with suspicious LN enlargement underwent therapeutic LN dissection in addition to total thyroidectomy.

Demographic data, pathologic characteristics including tumor size, ETE, resection margin involvement, coexisting Hashimoto thyroiditis, LN metastasis, multifocality, and adjuvant radioiodine treatment were recorded. On the histopathological examination, entire thyroid glands were serially sectioned and examined. The World Health Organization criteria for PTC variants and the American Joint Committee on Cancer 7th edition were used for Tumor–Node–Metastasis (TNM) staging. Follow-up period and recurrence status were also collected and analyzed. The primary outcome measure was the recurrence-free survival (RFS).

To minimize selection bias and possible confounding effects, we performed 1:1 propensity score matching [20]. A propensity score measures the probability that a patient would have been treated using a covariates score. Thus, propensity score matching balances the covariates and increases the comparability between the patients with multifocal tumors and those with unifocal PTC. We selected 3 factors that could affect recurrences as follows: ETE, LN metastasis, and resection margin involvement. SPSS Statistics version 23.0 (IBM Corp., Armonk, NY, USA) was used for data analyses. Comparison of continuous data was performed using Student’s t-tests. Dichotomous data were compared using chi-squared tests. RFS were assessed by using the log rank test and Kaplan–Meier plots. Cox proportional-hazards regression analysis was used to evaluate the relationship between prognostic factors and recurrence. A *p*-value less than 0.05 was considered statistically significant.

## 3. Results

### 3.1. Clinicopathological Characteristics of 1249 PTC Patients

The baseline characteristics of the included patients are summarized in Table 1. Mean age was 47.4 ± 11.4 years at the time of surgery and 1095 (87.7%) were women. The mean follow-up period was 5.5 ± 2.7 years (range, 1.0–11.1 years). Most patients (93.5%) had classical subtype of PTCs, while the remaining 81 patients with PTC variants included 53 patients with follicular variants including 7 encapsulated forms, 10 tall cell variants, 9 encapsulated variants, 4 oncocytic variants, 3 diffuse sclerosing variants, 1 columnar cell variant, and 1 hobnail variant.

Of the 1249 patients enrolled, 487 patients (39.0%) had multifocal PTCs and 762 (61.0%) had unifocal tumor. Thyroid cancer in patients with multifocal PTCs showed a higher rate of ETE (67.3% vs. 57.3%; *p* < 0.001) and a microscopic resection margin involvement (4.5% vs. 2.4%; *p* = 0.035) than in patients with unifocal tumor (Table 1). LN metastasis was also more common in the multifocality group than in the unifocality group (*p* = 0.007). Distant metastasis was not observed in all patients. Other clinicopathological factors, including age, sex, and tumor size, showed no significant differences between the groups.

**Figure 1:**
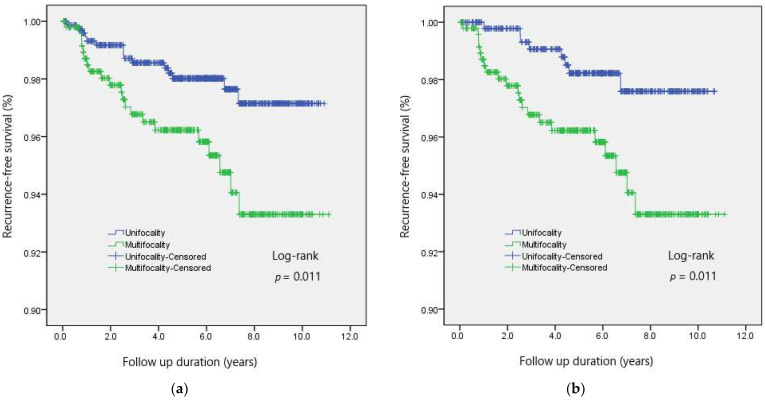
Recurrence-free survival according to the multifocality in patients with PTC, (**a**) before and (**b**) after propensity score matching.

Recurrences were found in 21 patients (4.3%) in patients with multifocal PTCs, and 15 patients (2.0%) with unifocal tumor developed recurrence (*p* = 0.013). A log-rank test indicated that 10-year RFS was significantly lower in the multifocality group (93.3% vs. 97.1%; *p* = 0.011) than in the unifocality group (Figure 1a).

### 3.2. Comparison of Recurrence Rates in the Matched Cohorts

As LN metastasis or resection margin involvement could affect the risk of recurrence, we performed 1:1 propensity score matching and yielded 487 matched pairs. Table 2 shows the clinicopathological comparison between the multifocality group and the 1:1 matched unifocality group. The matched cohorts did not differ in terms of clinicopathological features including microscopic ETE, margin involvement, and LN metastasis.

The overall recurrence rate was higher in the multifocality group than in the matched unifocality group (4.3% vs. 1.6%; *p* = 0.014), after adjustment of potential confounders. The 10-year RFS was also lower in the multifocality group (93.3% vs. 97.6%; *p* = 0.011) than in the matched group (Figure 1b).

### 3.3. Predictive Factors of Poor RFS in Patients with PTC

Univariate Cox proportional-hazards model indicated that male sex (HR 2.974, 95% CI 1.433–6.170), tumor size (HR 2.340, 95% CI 1.833–2.987), microscopic ETE (HR 2.708, 95% CI 1.186–6.182), LN metastasis (HR for N1a 3.858, 95% CI 1.587–9.380; HR for N1b 12.704, 95% CI 5.066–31.857), ^131^I remnant ablation (HR 6.512, 95% CI 2.709–15.656), and multifocality (HR 2.294, 95% CI 1.183–4.451) were significantly associated with the recurrence (Table 3). Male sex (HR 2.185, 95% CI 1.047–4.559), tumor size (HR 1.806, 95% CI 1.337–2.441), N1b LN metastasis (HR 3.603, 95% CI 1.207–10.757), and multifocality (HR 1.986, 95% CI 1.015–3.888) retained statistical significance in multivariate analysis.

## 4. Discussion

The present study demonstrates that multifocal PTCs are associated with a higher risk of recurrence. Multifocality is defined as the simultaneous presence of two or more anatomically separated foci within the thyroid gland [21]. Multifocal PTCs may result from intrathyroidal spread of original tumor or from multicentric independent PTCs [21]. The prevalence of multifocality in PTC ranges from 7.2 to 60.1% of the cases in the recent series [22,23]. The occurrence of multifocality varies according to the epidemiological and environmental factors [6]. Development of multifocal tumors can be associated with radiation, genetic disorders, or a family history of thyroid cancer [24,25]. A BRAF mutation also plays a role in inducing multifocality [26,27]. Furthermore, obese and overweight patients had a higher risk of multifocality [28].

Multifocality is associated with some high-risk features for the progression of PTC [7,8,9]. We demonstrated that multifocality was associated with higher ETE, LN metastasis, and microscopic resection margin involvement. Feng et al. showed that patients with multifocal PTCs had higher risk of large tumor size, ETE, vascular invasion, and LN metastasis [29]. Other researchers further indicated that multifocality was related with aggressive histologic subtype or higher ATA risk of recurrence [14,15]. A previous meta-analysis also suggested that multifocality was associated with an increased risk of tumor size >1 cm, ETE, and LN metastasis [13]. Hence, more radical treatments, including total thyroidectomy and radioactive iodine ablation, were commonly applied to patients with multifocal PTCs [6,7,30].

There is a controversy as to whether multifocality itself increases the risk of recurrence. Multifocality-associated high-risk features including ETE, LN metastasis, and margin involvement can affect the risk of recurrences. Previous studies further demonstrated that the impact of multifocality might be different according to the primary tumor size. In the present study, therefore, we performed propensity score matching to adjust potential confounders for minimizing biases. After propensity score matching, the overall recurrence rate was still higher in the multifocality group (4.3% vs. 1.6%; *p* = 0.014) than in the unifocality group. Survival analysis also indicated that patients with multifocal PTCs had a 1.986-fold higher risk of developing recurrences than those with unifocal tumors. Further validation studies may be helpful to confirm the impact of multifocality.

In the present study, male sex, tumor size, and N1b LN metastasis significantly increased the risk of recurrence, respectively. Data from the Canadian Collaborative Network for Cancer of the Thyroid indicated that men were at greater risk for recurrence than women (HR 2.31, 95% CI 1.48–3.60) [31]. Tumor size also has been widely accepted as a risk factor for recurrence in various risk stratification system, including AMES, AGES, and MACIS score [32]. N1b LN metastasis has been further recognized as a predictive factor for recurrence [33]. A meta-analysis suggested that a tumor size over 2 cm (OR 2.69, 95% CI 2.06–3.50) and LN metastasis (OR 3.24, 95% CI 2.61–4.02) were predictive factors for recurrence [34]. Wang et al. demonstrated that male sex, tumor size, and LN metastasis were associated with tumor recurrence of PTC in their large, multicenter study [12]. Our results are consistent with these previous reports.

This study has some limitations. First, our study was a retrospective cohort study, which was prone to a selection bias. Patient selection for receiving radioactive iodine ablation might be influenced by various factors and result in the difference of tumor recurrence. Second, we did not consider a family history or genetic mutation including BRAF mutation. Familial nonmedullary thyroid carcinoma can be more aggressive than the sporadic form [24,25]. However, because of the lack of data, we cannot evaluate the effect of family history or genetic mutation in the present study. Validation for the impact of multifocality is required in patients with family history or BRAF mutation. Third, we did not investigate the long-term prognosis including mortality. The mean follow-up period of 5.5 years was not sufficient for evaluating cancer-specific survival. Last, it is unclear whether patients with multifocal PTCs require aggressive treatment, although we demonstrated that multifocality increased the risk of recurrence. Further comparative studies are warranted to address these issues.

## 5. Conclusions

Multifocality increased the risk of recurrence in patients with PTC. Patients with multifocal PTCs may need judicious treatment and follow-up approaches.

## Figures and Tables

**Table 1:** Comparison of clinicopathological characteristics between patients with multifocal PTCs and those with unifocal tumors.

Characteristics	Multifocal (*n* = 487)	Unifocal (*n* = 762)	*p*-Value
Age (years)	47.8 ± 11.5	47.1 ± 11.4	0.330
Female sex	425 (87.3%)	670 (87.9%)	0.730
Pathologic characteristics			
Subtype			0.244
Classical	460 (94.5%)	708 (92.9%)	
Follicular	20 (4.1%)	33 (4.3%)	
Tall cell	3 (0.6%)	7 (0.9%)	
Encapsulated	1 (0.2%)	8 (1.0%)	
Oncocytic	0 (0.0%)	4 (0.5%)	
Diffuse sclerosing	2 (0.4%)	1 (0.1%)	
Hobnail	1 (0.2%)	0 (0.0%)	
Columnar	0 (0.0%)	1 (0.1%)	
Tumor size (cm)	1.0 ± 0.7	1.0 ± 0.7	0.133
Microscopic ETE	327 (67.3%)	436 (57.3%)	<0.001
Lymphovascular invasion	10 (2.1%)	17 (2.2%)	0.833
Perineural invasion	1 (0.2%)	2 (0.3%)	0.841
LN metastasis			0.007
N0	258 (53.0%)	463 (60.8%)	
N1a	171 (35.1%)	240 (31.5%)	
N1b	58 (11.9%)	59 (7.7%)	
Margin involvement	22 (4.5%)	18 (2.4%)	0.035
Coexisting HT	130 (26.7%)	214 (28.1%)	0.592
Postoperative management			
^131^I remnant ablation	227 (46.6%)	313 (41.1%)	0.054
^131^I dose (mCi)	135.4 ± 38.2	134.6 ± 31.9	0.790
Follow-up period (years)	5.3 ± 2.8	5.6 ± 2.6	0.042
Recurrence	21 (4.3%)	15 (2.0%)	0.016

PTC, papillary thyroid carcinoma; ETE, extrathyroidal extension; LN, lymph node; HT, Hashimoto thyroiditis.

**Table 2:** Comparison of clinicopathological characteristics between patients with multifocal PTCs and those with unifocal tumor after propensity score matching.

Characteristics	Multifocal (*n* = 487)	Unifocal (*n* = 487)	*p*-Value
Age (years)	47.8 ± 11.5	47.0 ± 12.0	0.279
Female sex	425 (87.3%)	426 (87.5%)	0.923
Pathologic characteristics			
Subtype			0.996
Classical	460 (94.5%)	460 (92.9%)	
Follicular	20 (4.1%)	21 (4.3%)	
Tall cell	3 (0.6%)	3 (0.9%)	
Encapsulated	1 (0.2%)	1 (1.0%)	
Oncocytic	0 (0.0%)	0 (0.5%)	
Diffuse sclerosing	2 (0.4%)	1 (0.1%)	
Hobnail	1 (0.2%)	0 (0.0%)	
Columnar	0 (0.0%)	0 (0.1%)	
Tumor size (cm)	1.0 ± 0.7	1.0 ± 0.7	0.864
Microscopic ETE	327 (67.3%)	319 (65.6%)	0.587
Lymphovascular invasion	10 (2.1%)	12 (2.5%)	0.666
Perineural invasion	1 (0.2%)	1 (0.2%)	1.000
LN metastasis			0.995
N0	258 (53.0%)	257 (52.8%)	
N1a	171 (35.1%)	171 (35.1%)	
N1b	58 (11.9%)	59 (12.1%)	
Margin involvement	22 (4.5%)	18 (3.7%)	0.518
Coexisting HT	130 (26.7%)	127 (26.1%)	0.827
Postoperative management			
^131^I remnant ablation	227 (46.6%)	236 (48.6%)	0.543
^131^I dose (mCi)	135.4 ± 38.2	136.3 ± 32.7	0.786
Follow-up period (years)	5.3 ± 2.8	5.6 ± 2.6	0.089
Recurrence	21 (4.3%)	8 (1.6%)	0.014

PTC, papillary thyroid carcinoma; ETE, extrathyroidal extension; LN, lymph node; HT, Hashimoto thyroiditis.

**Table 3:** Comparison of clinicopathological characteristics between patients with multifocal PTCs and those with unifocal tumor after propensity score matching.

Characteristics	Univariate Analysis		Multivariate Analysis	
	HR (95% CI)	*p*-Value	HR (95% CI)	*p*-Value
Age (years)	0.982 (0.952–1.012)	0.239		
Male sex	2.974 (1.433–6.170)	0.003	2.185 (1.047–4.559)	0.037
Tumor size (cm)	2.340 (1.833–2.987)	<0.001	1.806 (1.337–2.441)	<0.001
Microscopic ETE	2.708 (1.186–6.182)	0.018	1.311 (0.551–3.122)	0.541
LN metastasis				
N1a	3.858 (1.587–9.380)	0.003	1.859 (0.677–5.102)	0.229
N1b	12.704 (5.066–31.857)	<0.001	3.603 (1.207–10.757)	0.022
Margin involvement	2.071 (0.497–8.628)	0.317		
Hashimoto thyroiditis	0.643 (0.282–1.467)	0.294		
^131^I remnant ablation	6.512 (2.709–15.656)	<0.001	2.345 (0.807–6.811)	0.117
Multifocality	2.294 (1.183–4.451)	0.014	1.986 (1.015–3.888)	0.045

HR, hazard ratio; CI, confidence interval; ETE, extrathyroidal extension; LN, lymph node.

## Data Availability

The data presented in this study are available on request from the corresponding author. The data are not publicly available due to institutional policy.
